# CaveCrawler: an interactive analysis suite for cavefish bioinformatics

**DOI:** 10.1093/g3journal/jkac132

**Published:** 2022-06-16

**Authors:** Annabel Perry, Suzanne E McGaugh, Alex C Keene, Heath Blackmon

**Affiliations:** Department of Biology, Texas A&M University, College Station, TX 77843, USA; Department of Ecology, Evolution, and Behavior, University of Minnesota, Saint Paul, MN 55108, USA; Department of Biology, Texas A&M University, College Station, TX 77843, USA; Department of Biology, Texas A&M University, College Station, TX 77843, USA

**Keywords:** *Astyanax mexicanus*, database, genomics, model organism, *arpin*

## Abstract

The growing use of genomics in diverse organisms provides the basis for identifying genomic and transcriptional differences across species and experimental conditions. Databases containing genomic and functional data have played critical roles in the development of numerous genetic models but most emerging models lack such databases. The Mexican tetra, *Astyanax mexicanus* exists as 2 morphs: surface-dwelling and cave-dwelling. There exist at least 30 cave populations, providing a system to study convergent evolution. We have generated a web-based analysis suite that integrates datasets from different studies to identify how gene transcription and genetic markers of selection differ between populations and across experimental contexts. Results of diverse studies can be analyzed in conjunction with other genetic data (e.g. Gene Ontology information), to enable biological inference from cross-study patterns and identify future avenues of research. Furthermore, the framework that we have built for *A. mexicanus* can be adapted for other emerging model systems.

## Introduction

The reduced cost and increased efficiency of sequencing has led to enormous growth in the application of sequencing approaches to study diverse biological processes. In previous decades, these approaches were predominantly performed on a small number of genetically amenable model organisms including *Caenorhabditis elegans*, *Drosophila melanogaster*, zebrafish, and mouse. Model organism databases have been generated for each of these species, providing critical resources that decrease access barriers to genomic and phenotypic data (Harris *et al.* 2020; Howe, Ramachandran, *et al.* 2021; Larkin *et al.* 2021). Recently, there has been increased application of genomic and molecular approaches to nonstandard model systems, as these model systems may enable comparative evolutionary studies not possible in traditional systems (Juntti 2019). However, a lack of databases and analytic tools for many of these emerging model organisms impedes analysis of genomic data collected across different studies.

The Mexican tetra, *Astyanax mexicanus*, is an emerging model system to study the convergent evolution of diverse biological traits. These fish are comprised of a single population of river dwelling surface fish and at least 30 cavefish populations of the same species (McGaugh, Kowalko, *et al.* 2020). At least 2 evolutionary origins of the cavefish phenotype have occurred among *A. mexicanus* cavefish populations, resulting in numerous morphological, behavioral, and physiological differences from their surface conspecifics (Gross 2012; Jeffery 2020). These fish can be efficiently reared in laboratories, allowing for the application of transgenic and gene-editing approaches (Klaassen *et al.* 2018). There is a rapidly growing focus on genomic data that compares cave and surface populations. Current genomic data includes fully assembled genomes for surface and cave populations, population genetic resequencing, and transcriptomic data across different experimental conditions (Herman *et al.* 2018; Warren *et al.* 2021). The development of a database that compiles the growing number of genomics data across different experimental contexts would provide a valuable resource for accessing and analyzing this information.

The Shiny package in R offers a method to produce powerful community web resources that can go far beyond traditional repositories of data (Chang *et al.* 2021). Shiny databases enable researchers to incorporate the statistical analysis and data visualization capabilities of the R programming language into a reactive database that also functions as a community data repository. The combination of these tools allows users to sift through vast amounts of data, enabling novel discoveries (Blackmon and Demuth 2015). The generation of a Shiny database for comparative models of evolution could combine data across populations and studies. The flexibility of these systems and intrinsic analysis capabilities allows for direct comparisons of genetic data from disparate sources. Here, we generated a Shiny database, CaveCrawler, which combines population genetics and transcriptomic data from multiple Mexican tetra populations and leverages Gene Ontology (GO) term information to enable unique biological inferences from cross-study patterns. We demonstrate that the analysis features of this program can identify genes that are implicated in evolutionary processes across populations of *A. mexicanus*, using different methodologies from different studies.

## Methods

The CaveCrawler genetics inference tool acts as a reactive repository for transcription, GO, population genetics, and annotated genome data acquired from different studies in *A. mexicanus*, including those using reference genomes for surface and Pachón cavefish (McGaugh *et al.* 2014; Warren *et al.* 2021). With a highly accessible web interface, CaveCrawler enables researchers to search for data on genes-of-interest, find genes whose transcriptional levels match defined criteria, find genes which fit desired population genetics parameters, and also identify genes associated with cellular components, molecular functions, and biological processes.

### CaveCrawler modules

**Fig. 1.**
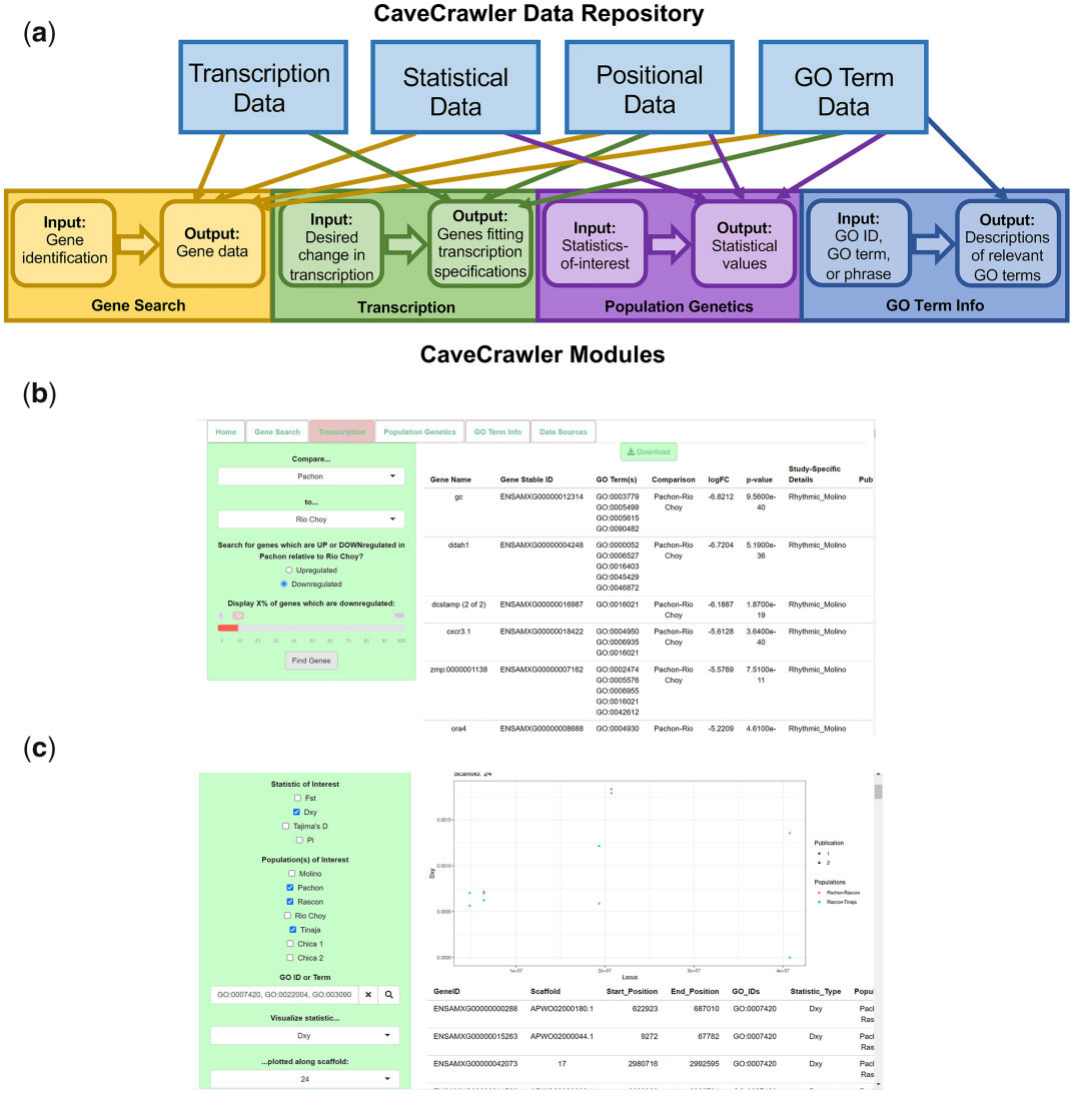
Design and web interface for CaveCrawler. a) The repository and module framework for the CaveCrawler model organism genomics database. Lines show the connections between different types of data stored in the repository and the user modules that draw on each data type. b) Example of the Transcription module with the results of searching for top 10% of genes that are downregulated in Pachón relative to Río Choy surface fish. c) Example of the Population Genetics module with the results of searching for the Pachón-Rascon surface fish and Rascon-Tinaja *d*_XY_ values of genes associated with brain development GO IDs and visualizing these values on Scaffold 24.

The CaveCrawler framework utilizes a bifurcated design with an underlying data repository and a collection of user interface modules (Fig. 1). This tool currently has 6 user modules: Home, Gene Search, Transcription, Population Genetics, GO Term Info, and Data Sources. The first 5 modules draw on different elements of the underlying data repository, while the Data Sources module describes the publications and methods of collection for each underlying dataset. This bifurcated design facilitates simple updates to the repository which then are immediately populated into changes in the functionality and results produced by the modules that draw on the updated repositories. Similarly, new modules can be added at any time to take advantage of new types of analyses or new data types. The Home module houses general information about *A. mexicanus* and about CaveCrawler’s functionality, as well brief instructions for contributing data.

The Gene Search module enables the user to search for data associated with genes-of-interest and also to identify genes associated with GO terms-of-interest. In this module, the user inputs a single gene stable ID, a single GO term, or a comma-separated list of genes. The module outputs a downloadable table describing all genes associated with the inputs and the positional, transcription, and population genetics data associated with each of the genes. The output also indicates whether a statistic or piece of transcriptional data is not present for each gene-of-interest. Therefore, this module concatenates data from disparate sources into a single analysis output, enabling the user to efficiently search for existing data and identify experiments which have yet to be conducted on their genes-of-interest.

The Transcription module enables the user to identify genes which differ in transcription level between groups. Here, the user first inputs the groups they would like to compare. The user may either compare an experimental group to a control group or compare one population to another population. The user then specifies whether they would like to see genes which are up or downregulated in the first group compared with the second and the % change in transcription level between groups. The module then produces a downloadable output table of genes fitting the specified transcription patterns.

The Population Genetics module enables the user to access population genetics statistics, such as π, Tajima’s *D*, *d*_XY_, and *F*_ST_. This module has 2 options for accessing population genomics data. In the first option, the user provides GO terms and the module outputs and visualizes the statistical values of all genes associated with those GO terms. The second approach enables the user to search for transcriptional or genomic values calculated in different studies.

In the GO term search submodule of the Population Genetics module, the user inputs GO information, statistics-of-interest, and populations-of-interest. For the GO information, the user can input either a single GO ID, a comma-separated list of GO IDs, or a phrase associated with the target GO term. The module outputs a downloadable table describing all values of the population-specific statistics-of-interest for the genes associated with the indicated GO term(s). If any of the statistics-of-interest require pairwise comparisons between populations, the module will output pairwise statistics for each possible pairing of input populations. On this submodule, the user may also input a statistic and a scaffold and CaveCrawler will plot the statistical values of each GO term-associated gene which falls on that scaffold. The GO term function of the Population Genetics module thus enables the user to access and visualize population genomics statistics for a GO term of interest.

The outlier function of the Population Genetics module consists of 2 approaches for pulling outlier genes from combined datasets. One approach enables the user to identify a specified number of genes which have the most extreme values for an indicated statistic, while the other approach enables the user to identify all genes with specific statistics above or below a specified threshold value. In the gene number approach, the user must specify the number of genes and must specify whether they would like to see the top or bottom quantile. CaveCrawler then outputs a table describing the specified number of genes with the most extreme values for the statistic-of-interest. In the statistical threshold approach, the user specifies a threshold statistical value and specifies whether they would like to see genes above or below this value. CaveCrawler outputs both a table and a distribution plot describing the genes which fall above or below this threshold.

Both outlier approaches require the user input a statistic-of-interest and population(s)-of-interest. If the statistic-of-interest is a 1 population statistic, such as π or Tajima’s *D*, both approaches will report outlier statistical values for all input populations. If the input statistic is a pairwise statistic, such as *F*_ST_ or *d*_XY_, both approaches will report outlier statistical values for all possible pairs of populations-of-interest. If a statistic value has yet to be collected for a population or population pair, CaveCrawler will output a warning about that statistic. Thus, the outlier function of the Population Genetics module enables users to not only identify outliers for a statistic-of-interest but also to identify populations for which a statistic-of-interest has yet to be collected.

The GO Term Info module enables users to access descriptions of GO IDs. This function helps users identify GO IDs they should search for in the Population Genetics module and helps them make sense of transcription and outlier queries. On this module, the user may input a single GO ID, comma-separated list of GO IDs, or a phrase-of-interest, such as “sleep.” CaveCrawler searches data from the official GO Consortium databank (version displayed in module) and outputs descriptions of all input GO IDs or GO IDs relevant to the input phrase. In addition, CaveCrawler reports all GO IDs which occur hierarchically beneath these IDs. The GO Term Info module thus enables researchers to investigate the broader biological impact of transcription and diversity data relevant to their genes-of-interest.

### The data repository

CaveCrawler currently pools data from multiple publications and authors can request that their own data be integrated into CaveCrawler’s repository. As of publication, CaveCrawler’s data bank includes transcriptional datasets (Mack *et al.* 2021; Moran *et al.* 2022), population genetics datasets (Herman *et al.* 2018; McGaugh, Passow, *et al.* 2020), GO data from UniProt and the Gene Ontology Consortium (Ashburner *et al.* 2000; The Gene Ontology Consortium 2021; The UniProt Consortium 2021), and genome architecture data from Ensembl Genome Browser, release 104 (Howe, Achuthan, *et al.* 2021). The specific datasets which CaveCrawler draws data from are described in Table 1.

**Table 1.** Locations of specific datasets used by CaveCrawler modules.

Publication	Specific dataset(s)	Modules
Mack *et al.* (2021)	Supplementary File 1, BaseShifts sheet	Gene Search Transcription
Moran *et al.* (2022)	Extended Data Supplementary Table 9	Gene Search Population Genetics
McGaugh, Passow, *et al.* (2020)	McGaugh.et.al.2019.Sleep.Dep.Sup.Mat.xlsx Supplementary Tables 2–5	Gene Search Transcription
Herman *et al.* (2018)	Supplementary Tables 11 and 13	Gene Search Population Genetics

The first column describes the publication from which the data came, the middle column lists the file(s) from that publication which were used in CaveCrawler, and the rightmost column lists the CaveCrawler modules which integrate the indicated data. These publications are also cited in the References of this paper and on the Data Sources module of the CaveCrawler GUI.

CaveCrawler’s Transcription and Gene Search modules currently draw upon datasets that describe genes whose transcription levels changed significantly in response to sleep deprivation in *A. mexicanus* (McGaugh, Passow, *et al.* 2020). This dataset describes the log fold-change (logFC) and *P*-values for each of these genes in each *A. mexicanus* morph where the change in transcription was significant compared with controls of the same morph (McGaugh, Passow, *et al.* 2020). The surface fish genes for this dataset were originally mapped to *A. mexicanus* genome 1.02 (McGaugh, Passow, *et al.* 2020), but were remapped to *A. mexicanus* 2.0 prior to integration into CaveCrawler. The Transcription and Gene Search modules also access transcription data for circadian-related genes whose transcription is significantly different between morphs (Mack *et al.* 2021). The Transcription module has enough flexibility that new transcriptional data can be integrated. Thus, CaveCrawler could be used to analyze transcriptional changes in response to any experimental condition and between any 2 populations of *A. mexicanus.*

CaveCrawler’s Population Genetics and Gene Search modules currently integrate data from 2 studies describing signatures of selection in *A. mexicanus* (Herman *et al.* 2018; Moran *et al.* 2022). One of these studies calculated π and Tajima’s *D* values for the Pachón, Tinaja, Molino, Río Choy, and Rascón populations, as well as *F*_ST_ and *d*_XY_ values for each population pair (Herman *et al.* 2018). The other study describes *d*_XY_ values of all genes in 2 populations of the Chica morph, Pachón and Rascón, and Tinaja and Rascón (Moran *et al.* 2022). CaveCrawler itself does not calculate new population genetics statistics but instead integrates statistics calculated in previous studies. As with the Transcription module, the Population and Gene Search modules have enough flexibility that new data can be integrated.

The Gene Search, Transcription, and Population Genetics modules currently draw upon positional data obtained from Ensembl (Howe, Achuthan, *et al.* 2021). The genome assembly used in the current version is *A. mexicanus* 2.0, the most up-to-date genome assembly for this species (Warren *et al.* 2021). All of CaveCrawler’s modules utilize GO term information from UniProtKB, 2021 February 2 release and from the Gene Ontology Consortium, 2021 September release (Ashburner *et al.* 2000; The Gene Ontology Consortium 2021; The UniProt Consortium 2021).

Though CaveCrawler already integrates data from numerous disparate sources, enabling powerful cross-study comparisons of genetic data, CaveCrawler’s data repository is not static. The CaveCrawler website includes instructions for data submission and the power and insights possible with this resource will grow as the repository of data on which it draws grows. CaveCrawler’s data repository will be updated annually in July.

## Results

The CaveCrawler analysis suite consists of multiple tools for comparing datasets that allow for identification of genetic differences between populations of *A. mexicanus*. These tools have a wide range of applications, including rapid candidate gene identification and inference of population-level variation. Here, we present an example of how CaveCrawler can be used to answer biological questions.

### Rapid identification of candidate genes for empirical studies

Since CaveCrawler enables simultaneous cross-analysis of multiple studies, researchers can use CaveCrawler to find genes which are outliers for both transcription and population genetics statistics in a matter of minutes. These genes can then be analyzed in downstream studies, such as GO term analyses, to make biological inferences. Here, we identified genes which are transcriptionally dysregulated between cave and Río Choy morphs, then performed a GO term analysis to determine the biological function and cellular components with which these genes are associated. These genes could be used as candidates for future empirical studies, such as knockdown or knockout studies.

To examine genes that are both transcriptionally upregulated and harbor markers of selection, we first used CaveCrawler’s Population Genetics module to identify the *F*_ST_ values of all genes whose *F*_ST_ values were published in a recent population inference paper in the Mexican tetra (see Herman *et al.* 2018). Then, we used the “Gene Search” module to identify the transcription data for each of the 1,140 genes identified by the Population Genetics module (see Supplementary Materials). Pairwise *F*_ST_ measures how dissimilar a DNA sequence is between 2 groups relative to diversity within the groups, and logFC is the log fold change in mRNA transcription between 2 groups (Charlesworth 1998; Mack *et al.* 2021). Of the genes for which both *F*_ST_ and logFC had been calculated by previous studies, there were 83 for which *F*_ST_ outlier status had been determined by a previous study which defined *F*_ST_ outliers as genes whose *F*_ST_ values were in the lowest 5% of divergence (Herman *et al.* 2018). Gene names, logFC, transcription *P*-values, and *F*_ST_ values for all 83 genes are available in Supplementary Materials.

**Fig. 2.**
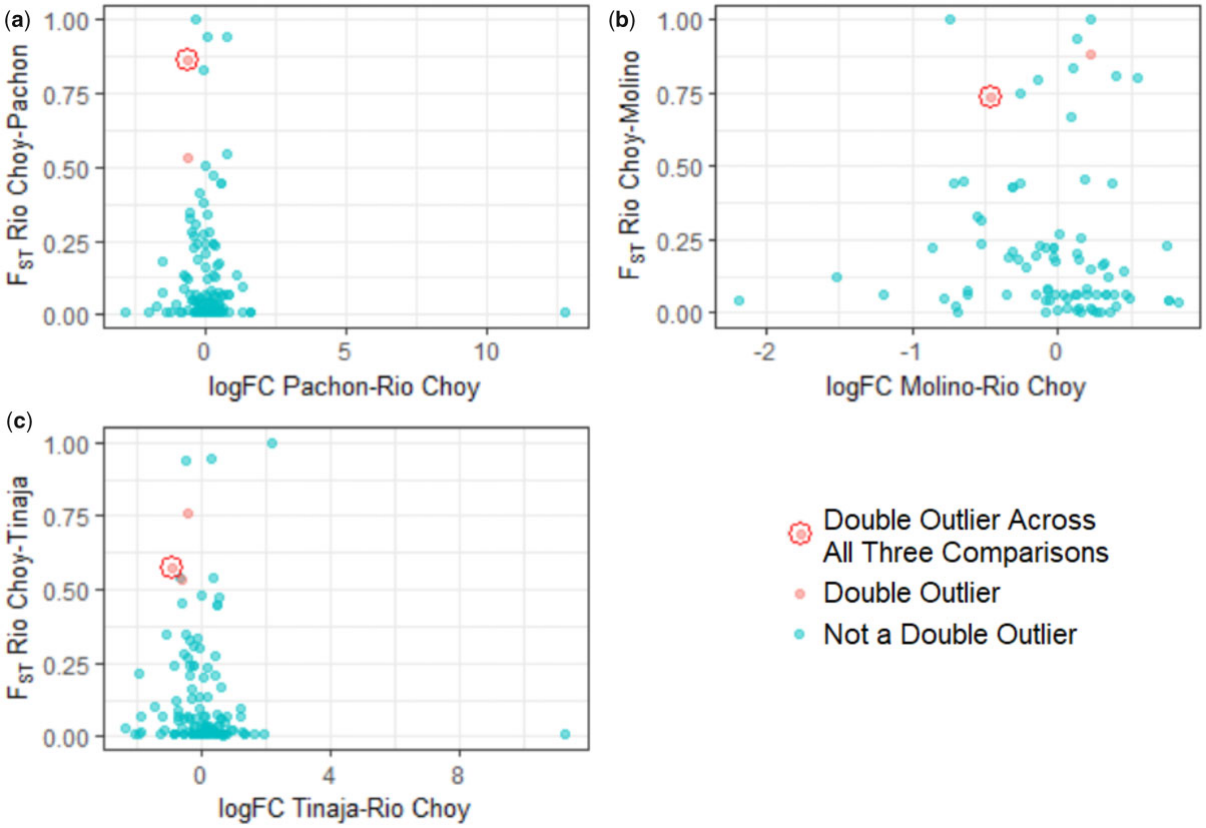
Overlap between FST values and logFC values across multiple studies. Plots of cave-specific FST vs logFC values for all 83 genes which CaveCrawler found to have FST values, logFC values, and FST outlier (lowest 5% of divergence) designations. Double-outliers for the cave-Río Choy comparison indicated by the axes are colored in red, while the gene (*arpin*) which was a double-outlier across in all 3 cave-Río Choy comparisons is encircled in red. Transcription data comes from a study describing differences in regulation of circadian-related genes between morphs (Mack *et al.* 2021). a) Pachón vs Río Choy; b) Molino vs Río Choy; and c) Tinaja vs Río Choy.

For each cave-Río Choy surface comparison, we then identified the genes which were both significantly differentially expressed for circadian regulation (logFC *P*-value <0.05) between Río Choy and the corresponding cave population and were identified by a previous study to be *F*_ST_ outliers for the same population pairing (Herman *et al.* 2018). These genes, which were both transcriptional and *F*_ST_ outliers, will henceforth be referred to as double outliers. We found one gene, *arpin*, which was a double outlier in all 3 cave-Río Choy pairings (Table 2 and Fig. 2), one, *cyp26a1*, which was a double outlier for both Pachón-Río Choy and for Tinaja-Río Choy (Table 2; Fig. 2, a and c), one, *si: dkeyp-84f3.5*, which was a double outlier for Molino-Río Choy only (Table 2 and Fig. 2b), and one, *socs6b*, which was a double outlier for Tinaja-Río Choy only (Table 2 and Fig. 2c).

**Table 2.** Genes identified as outliers for *F*_ST_ and transcriptional regulation over the circadian cycle between surface fish and 3 different cavefish populations.

Gene name	Gene description	Comparison	Double outlier	*F*_ST_	logFC	*P*-value for logFC
***si:dkeyp-84f3.5***	NA	Pachón vs Río Choy	No	0.2780	0.1390	0.0558
		Molino vs Río Choy	Yes	0.8828	0.2253	0.0035
		Tinaja vs Río Choy	No	0.3003	−0.0121	0.8625
***socs6b***	Suppressor of cytokine signaling 6b (Source: ZFIN;Acc:ZDB-GENE-030131-1670)	Pachón vs Río Choy	No	0.8266	−0.0433	0.8329
		Molino vs Río Choy	No	0.8363	0.1074	0.5477
		Tinaja vs Río Choy	Yes	0.7565	−0.4082	0.0156
***cyp26a1***	Cytochrome P450, family 26, subfamily A, polypeptide 1 (Source: ZFIN;Acc:ZDB-GENE-990415-44)	Pachón vs Río Choy	Yes	0.5323	−0.6150	0.0074
		Molino vs Río Choy	No	0.7924	−0.1403	0.5548
		Tinaja vs Río Choy	Yes	0.5365	−0.5950	0.0097
***arpin***	Actin-related protein 2/3 complex inhibitor (Source: HGNC Symbol;Acc:HGNC:28782)	Pachón vs Río Choy	Yes	0.8612	−0.5847	0.0004
		Molino vs Río Choy	Yes	0.7343	−0.4623	0.0001
		Tinaja vs Río Choy	Yes	0.5762	−0.8816	1.33E−10

*F*_ST_ and logFC values for all genes which were found to be outliers for both *F*_ST_ and logFC in at least 1 cave-Río Choy comparison.

We performed a GO term analysis on *arpin* to identify any biological process, molecular function, or cellular component associated with this double outlier. We found *arpin* to be associated with the biological process GO ID, GO:0051126 and the cellular component GO IDs, GO:0016021 and GO:0030027, which correspond to “negative regulation of actin nucleation,” “integral component of membrane,” and “lamellipodium,” respectively. To calculate the likelihood of sampling an *A. mexicanus* gene associated with GO:0051126 by chance, we performed a Monte Carlo simulation for 1 000 000 iterations and calculate an empirical *P*-value of 2.8e−05. We performed another Monte Carlo to find the likelihood of sampling GO:0016021 and GO:0030027 by chance, obtaining an empirical *P*-value of 4.4e−05. Thus, we used CaveCrawler to rapidly discover that genes that harbor markers of selection and are differentially expressed in cave populations across the circadian cycle.

As shown by this example, the CaveCrawler analysis suite can be used for a variety of investigations in the Mexican tetra. In minutes, CaveCrawler can combine statistics from multiple studies and leverage GO terms to make novel inferences about evolutionary forces acting within a population. CaveCrawler is accessible via internet search engines as cavecrawler.org and through the following url: www.cavecrawler.org.

## Discussion

Here, we describe a modular analysis suite for *A. mexicanus.* We have included a set of the genomics and transcriptional data that has been previously published (Herman *et al.* 2018; McGaugh, Passow *et al.* 2020; Howe, Achuthan *et al.* 2021; Mack *et al.* 2021; The UniProt Consortium 2021; Warren et al. 2021; Moran *et al.* 2022). In addition to these studies, transcriptional analysis across developmental timepoints, as well as single cell analysis of hypothalamus has been collected. These datasets, and others collected in the future can be added to this analysis suite. These data, in combination with assembled genomes for surface fish and Pachón cavefish provide a platform for gene discovery in this system. In addition, the modularity of this system allows it to be readily adapted for new data types or genomic analyses. We then demonstrated that this analysis suite can be used to combine data from disparate sources to discover novel patterns in the Mexican tetra genome.

As proof of principle, we performed an analysis for genes that contained markers of selection and transcriptional dysregulation across the circadian cycle. This analysis identified 4 genes that were significantly different. These genes are strong candidates for functional regulators of evolved differences in circadian behavior that have been widely studied in *A. mexicanus* and other species of cavefish (Teyke and Schaerer 1994; Beale *et al.* 2013; Moran *et al.* 2014; Ceinos *et al.* 2018; Mack *et al.* 2021). The gene *arpin*, a negative regulator of actin, is of particular interest because it is identified as harboring markers of selection and differential expression across all 3 cavefish populations included in this study. Actin dynamics have been implicated as targets of circadian regulation for a number of processes including wound healing, immune function and neural plasticity (Petsakou *et al.* 2015; Hoyle *et al.* 2017; Kitchen *et al.* 2020). Therefore, it is possible that multiple populations of cavefish have converged on changes in actin regulation that account for loss of behavioral and transcriptional rhythms (Beale *et al.* 2013; Mack *et al.* 2021).

Currently, annotated genomes exist for both surface fish and Pachón cavefish (McGaugh *et al.* 2014). The Pachón genome is the original genome to be sequenced using 100 bp paired-end reads, resulting in significant fragmentation. More recently, a surface fish genome was developed with long-read sequencing technology that provides improved resolution and more accurate mapping of QTL markers (Warren *et al.* 2021). The population genetics data included in this study use the Pachón genome, and it is possible that additional, or different genes would be identified using the surface fish genome. Therefore, reanalyzing previously acquired data to the Pachón reference genome using surface fish may identify novel genes associated with markers of selection. In addition, the Data Sources module of CaveCrawler will state which reference genome was used to acquire data.

Shiny has been widely applied to develop a range of public databases that offer interactive data visualization and access (Blackmon and Demuth 2015; Tree of Sex Consortium 2015; Reyes *et al.* 2019; Manchanda *et al.* 2020). However, to our knowledge, this is the first use of Shiny to create a public genomic database and analysis tool specific to a model organism. Traditionally public genomic databases, which are key to supporting model organism communities, have come with considerable cost in the form of computer programmers and hosting services (Oliver *et al.* 2016; Bellen *et al.* 2021). Perhaps one of the most valuable contributions that CaveCrawler can make is as a flexible framework that can be adopted by any model organism community. We have made the underlying code for this project publicly available under the GPL license.

The flexibility of CaveCrawler allows for the addition of modules that integrate genomics and guide efforts to validate gene function. In *A. mexicanus*, like many other models of evolution, studies identifying quantitative trait loci (QTL) have provided a basis for a growing genetic toolkit in *A. mexicanus* can be used for functional genomics experiments guided CaveCrawler (Casane and Rétaux 2016; Jeffery 2020). The flexibility of this system allows for the addition of modules to include these data and localize genes near existing QTL. These could help identify candidate genes for functional validations. For example, transgenesis, CRISPR-based transgenesis, and morpholinos have all been applied for functional validation of gene function (Elipot et al. 2014; Jaggard *et al.* 2018; Stahl, Jaggard, *et al.* 2019; Stahl, Peuß, *et al.* 2019). In addition, CRISPR-based screening approaches have been developed in zebrafish that allow for high throughput functional assessment of developmental and behavioral traits. This analysis suite will provide methodology for identifying genes for functional analysis.

## Data availability

All source code and example datasets are available in the GitHub repository: https://github.com/AnnabelPerry/CaveCrawler.

Supplemental material is available at *G3* online.

## Funding

This work was supported by an NIH NIGMS R35GM138098 to HB, NIH R01 1R01GM127872 to ACK, and SEM, NIH R21 NS122166 to ACK, and the Texas A&M University College of Science Undergraduate Research Opportunities Program to AP.

## Conflicts of interest

None declared.

## Supplementary Material

jkac132_Supplementary_Data
